# Novel self-amplificatory loop between T cells and tenocytes as a driver of chronicity in tendon disease

**DOI:** 10.1136/annrheumdis-2020-219335

**Published:** 2021-03-10

**Authors:** Emma Garcia-Melchor, Giacomo Cafaro, Lucy MacDonald, Lindsay A N Crowe, Shatakshi Sood, Michael McLean, Umberto G Fazzi, Iain B McInnes, Moeed Akbar, Neal L Millar

**Affiliations:** 1 Institute of Infection, Immunity and Inflammation, University of Glasgow College of Medical Veterinary and Life Sciences, Glasgow, UK; 2 Rheumatology Unit - Department of Medicine, University of Perugia, Perugia, Italy; 3 Department of Orthopaedic Surgery, Queen Elizabeth University Hospital, Glasgow, UK

**Keywords:** tendinopathy, T-lymphocyte subsets, inflammation

## Abstract

**Objectives:**

Increasing evidence suggests that inflammatory mechanisms play a key role in chronic tendon disease. After observing T cell signatures in human tendinopathy, we explored the interaction between T cells and tendon stromal cells or tenocytes to define their functional contribution to tissue remodelling and inflammation amplification and hence disease perpetuation.

**Methods:**

T cells were quantified and characterised in healthy and tendinopathic tissues by flow cytometry (FACS), imaging mass cytometry (IMC) and single cell RNA-seq. Tenocyte activation induced by conditioned media from primary damaged tendon or interleukin-1β was evaluated by qPCR. The role of tenocytes in regulating T cell migration was interrogated in a standard transwell membrane system. T cell activation (cell surface markers by FACS and cytokine release by ELISA) and changes in gene expression in tenocytes (qPCR) were assessed in cocultures of T cells and explanted tenocytes.

**Results:**

Significant quantitative differences were observed in healthy compared with tendinopathic tissues. IMC showed T cells in close proximity to tenocytes, suggesting tenocyte–T cell interactions. On activation, tenocytes upregulated inflammatory cytokines, chemokines and adhesion molecules implicated in T cell recruitment and activation. Conditioned media from activated tenocytes induced T cell migration and coculture of tenocytes with T cells resulted in reciprocal activation of T cells. In turn, these activated T cells upregulated production of inflammatory mediators in tenocytes, while increasing the pathogenic collagen 3/collagen 1 ratio.

**Conclusions:**

Interaction between T cells and tenocytes induces the expression of inflammatory cytokines/chemokines in tenocytes, alters collagen composition favouring collagen 3 and self-amplifies T cell activation via an auto-regulatory feedback loop. Selectively targeting this adaptive/stromal interface may provide novel translational strategies in the management of human tendon disorders.

**Key messages**

**What is already known about this subject?**

- Tendinopathy is a complex multi-faceted tendon pathology commonly associated with overuse. The potential roles of immune cells acting on resident tenocytes remain the source of some controversy while understanding these interactions on tendon homoeostasis is crucial in designing future targeted therapies.

**What does this study add?**

- Our study shows the spatial relationship of T cells and tenocytes in tendinopathy, an upregulation of inflammatory cytokines, chemokines and adhesion molecules in activated tenocytes after tendon damage, the effect of tenocytes on T-cell migration and activation and finally the impact of these activated T cells on the stromal compartment in the tendon, with the maintenance of an inflammatory response and changes in collagen 3/collagen 1 ratio.

**How might this impact on clinical practice or future developments?**

- Selectively targeting T cells in tendinopathy provides scope for novel translational strategies in the management of tendon disorders.

## Introduction

Tendinopathy is the broad term encompassing multifactorial tendon disorders clinically characterised by the presence of pain and functional limitation.^1^ Achilles, patellar and rotator cuff tendinopathies as well as epicondylitis are examples of this prevalent pathology that is responsible for up to 30% of general practice musculoskeletal consultations^2^ costing European Union healthcare systems in excess of €800 million annually.^3^

The paucity of effective treatments in tendon disorders partially reflects lack of understanding of its pathogenesis.^4^ Historically considered a degenerative pathology and therefore termed ‘tendinosis’, research conducted during the last decade has revealed the presence of immune cells (mast cells, macrophages, T cells)^5–12^ and inflammatory cytokines (interleukin (IL)-6, IL-15, IL-17, IL-18, IL-33, tumour necrosis factor alpha (TNF-α))^10 11 13 14^ in tendinopathic tissues. Recognition of this inflammatory component has important clinical implications, as these pathways provide therapeutic potential that may avoid chronicity and long-term complications such as pain and tendon ruptures.

During the inflammatory process there is crosstalk between the stromal compartment and infiltrating immune cells that can determine the fate of tissue in terms of repair or degeneration. In particular, the relationship between T cells and stroma has been intensively studied in other rheumatic and musculoskeletal diseases, such as rheumatoid arthritis (RA). Chemokines involved in T cell recruitment are present in synovial tissues and synovial fluid from patients with RA^15^ and synovial fibroblast subsets promote T cell survival,^16–19^ activation^17 20^ and induce interferon (IFN)-γ, IL-17 and TNF-α production.^20 21^ In turn, T cells have reciprocal effects on synovial fibroblasts through upregulation of ICAM-1, IL-6, IL-8, IL-15, IL-18, MMP1, MMP3, PGE2, TNF-α and VCAM-1 in the later.^17 20–23^ These pathways support persistence of the inflammatory infiltrate, hence chronicity. However, few data exist regarding the relationship between tendon stromal cells, also known as tenocytes, and T cells and its role in the development of chronicity in tendinopathy.

The development of tendon inflammation focused in areas where tendons attach to bones, also known as enthesitis, is characteristic of spondyloarthritis (SpA).^24^ Mechanical stress in these areas has been proposed as a trigger for the development of an inflammatory response that further extends into the synovial compartment. Although the presence of T cells has been observed at the enthesis,^25^ less is known about which is the relationship between T cells and tenocytes and its role in the development and perpetuation of a chronic inflammatory response in SpA.

We hypothesise that the cross-talk between tenocytes and T cells may play a role in the development of chronicity in tendon inflammation through its effect on sustaining an immune response by promoting migration and activation of immune cells and importantly, through changes collagen 3, which has an impact in tendon biomechanics. Herein, we analysed the effect of tenocytes on migration and activation of T cells and in turn the reciprocal effect of activated T cells on tenocytes. We describe a hitherto unrecognised self-amplificatory feedback loop in human tendon disease.

## Methods

### Tissue collection and preparation

Supraspinatus and subscapularis tendon samples were collected from patients with rotator cuff tears undergoing shoulder surgery for flow cytometry (FACS) analysis, mass cytometry imaging and single cell RNA-sequencing. Standardised patient demographics were obtained preoperatively and included age, sex, duration of shoulder symptoms experienced by the patient and the number of subacromial steroid injections (table 1). Patients who received two steroid injections had symptoms >6 months and had not had an injection at least 3 months prior to surgery. Samples of the subscapularis tendon were also collected from the same patients. Patients were only included if there was no clinically detectable evidence of subscapularis tendinopathy on a preoperative MRI scan as determined by a musculoskeletal radiologist or macroscopic damage to the subscapularis tendon at the time of arthroscopy as determined by the senior author (NLM)—by these criteria they represented a preclinical cohort. In this cohort, all patients fulfilled the following criteria^1^: a history of shoulder pain and dysfunction,^2^ no previous surgery on the affected shoulder,^3^ no radiographic sign of fracture of the shoulder and^4^ no history of RA or osteoarthritis. Hamstring tendons obtained at the time of routine anterior cruciate ligament (ACL) reconstruction were employed as an independent control group. Healthy human tenocytes were explanted from hamstring tendon of patients undergoing ACL reconstruction. Cells were maintained at 37°C in a humidified atmosphere of 5% CO_2_ for up to 28 days and then subcultured and trypsinised at subconfluency, using cells with less than four passages.

**Table 1:** Patient demographics for by imaging mass cytometry (IMC), flow cytometry (FACS) and single cell RNA-seq (scRNASeq)

Experiment	Tissue	Patients (n)	Mean age in years (range)	Sex (M:F)	Mean duration of symptoms in months (range)	Mean number of steroid injections
IMC	Supraspinatus tendinopathy (established tendinopathy)	5	58.6 (49–62)	2:03	6.4 (2–12)	1.3
FACS	Supraspinatus tendinopathy (established tendinopathy)	14	56.4 (39–65)	8:06	6.8 (3–11)	1.2
	Subscapularis Tendinopathy (early tendinopathy)	9	59.2 (46–63)	5:04	8.3 (4–13)	1.1
	Control tendon hamstrings tendon)	4	21.5 (19–25)	3:01	–	–
scRNASeq	Supraspinatus tendinopathy (established tendinopathy)	4	62 (58–68)	2:02	6.2 (2–12)	1
	Control tendon (hamstrings tendon)	5	25 (21–28)	3:02	–	–

### Immunophenotype of tendinopathic tissue

Tendon tissue was digested in 0.15 mg/mL Liberase TM (Sigma-Aldrich) in 10 mL RPMI, kept in constant rotation at 37°C for a maximum of 2 hours. Digested tissue was then filtered, and cells were stained with the following antibodies: anti-CD45 BV510 and anti-CD3 FITC, anti-CD19 APC, anti-CD64 APC-Cy7 and anti-CD117 PE (Biolegend). Samples were run on an LSRII flow cytometer (BD Biosciences) and results analysed with FlowJo software. Cells were initially gated on the forward and side scatter (online supplemental figure 1A), following identification of singlet cells, immune cells were gated on CD45+ events. Subsequently, macrophages (CD64+) and (CD3+) T Cells were identified, the negative population of both markers was then used to identify B cells (CD19+) and mast cells (CD117+). The number of events of each population was quantified and used to calculate the percentage of immune cells (CD45+) from the whole singlet cell population and the percentage of each immune cell population from the CD45+ immune cell subset (online supplemental figure 1B).

10.1136/annrheumdis-2020-219335.supp1 Supplementary data

### Histology and imaging mass cytometry

Samples were stained with H&E for determination of the degree of tendinopathy as assessed by a modified version of the Bonar score (grade 4=marked tendinopathy, grade 3=advanced tendinopathy, grade 2=moderate degeneration, grade 1=mild degeneration, grade 0=normal tendon). This included the presence or absence of oedema and degeneration together with the degree of fibroblast cellularity and chondroid metaplasia. Examples of H&E staining used in imaging mass cytometry (IMC) are provided in online supplemental figure 2A. Tissues were immediately fixed in 4% formalin for 4–6 hours and then embedded in paraffin. Sections were cut to 5 µM thickness using a Leica-LM microtome (Leica Microsystems, Germany) and placed onto Superfrost Ultra Plus glass slides (Gerhald Menzel, Germany). Samples were stained with the following antibodies: anti-human CD4 (EPR6855)—156Gd, anti-human CD68 (KP1)—159Tb, anti-human CD8a anti-human collagen type I (Polyclonal)—169Tm, anti-human CD3 (Polyclonal, C-Terminal)—170Er, anti-histone 3 (D1H2)—176Yb (all Fluidigm UK), using the IMC Staining Protocol for FFPE Sections from the manufacturer. Briefly, slides were baked for 2 hours at 60°C and then dewaxed in fresh xylene for 20 min followed by hydrating in descending grades of ethanol (100%, 95%, 80%, 70%), 5 min each. They were washed in MAXpar water (Fluidigm) and then placed into preheated antigen retrieval solution (pH9) (Agilent) and incubated for 30 min at 96°C. Slides were cooled and washed in Maxpar water and Maxpar PBS (Fluidigm) twice and blocked for 45 min with 3% BSA (Sigma-Aldrich), following staining with antibodies previously mentioned in 3%BSA overnight. After that slides were washed in Maxpar PBS and then Maxpar water prior to drying. Sections were then ablated on Hyperion Imaging System (Fluidigm) for analysis. Data were exported to MCD viewer (Fluidigm) and images converted to allow cell segmentation using Cell Profiler and Ilastik (Broad Institute). HistoCat (BodenMiller) and IMACytE (Leiden) was used for unsupervised clustering of cells (PhenoGraph function) and neighbouring cells were defined as those within four pixels.

10.1136/annrheumdis-2020-219335.supp2 Supplementary data

### Single-cell RNA-seq

Single-cell suspensions of cells were derived from freshly digested tendon biopsies following surgical excision, as previously described.^26^ Live cells were sorted using a FACS ARIA III. Isolated cells (13 561 cells from healthy and 38 040 cells from supraspinatus tendon tissue) were lysed and then RNA was reverse-transcribed and converted to cDNA libraries for RNA-seq analysis using a Chromium Controller and Chromium Single Cell 3′ v2 Reagent kit (10x Genomics) following the manufacturer’s protocol. Pooled libraries were used for sequencing on a HiSeq 4000 (Illumina) to a depth of ~30 000 reads per cell. Alignment of reads to the genome and generation of gene counts per cell were performed by Cell Ranger software (10x Genomics). 4080 cells from healthy and 22 004 cells from supraspinatus tendon tissue were sequenced. Quality control was performed on each sample and poor-quality cells were removed on the basis of number of genes expressed (<200), of unique molecular identifiers and percentage of mitochondrial reads mapped (>5%). Following this quality check (QC), we normalised and scaled the data using Seurat Package (Satija Lab) for all the cells (health k=3040, supraspinatus k=19 084). Then principal components analysis and high-quality cells were clustered using a graph-based routine implemented in Seurat R package and its integration method for multiple samples (Satija Lab). All cells from the tissue were clustered (online supplemental figure 1C), and immune cells were computationally isolated for further analysis (online supplemental figure 1D).

### Stimulation of tenocytes

Healthy hamstring tendon tissue from ACL reconstructions was cut in small pieces with a scalpel and transferred into flasks with complete RPMI (RPMI media supplemented with 10% FBS, penicillin 100 IU/mL, streptomycin 100 µg/mL), which were placed in an incubator at 37°C, 5% CO_2_. After 24 hours, 1 mL of this conditioned media obtained from damaging the tendon was stored at −20°C until use. Tenocytes were seeded into 24 well tissue culture plates (25 000 cells/well) and left to rest for 72 hours at 37°C, 5% CO_2_. On day 3, media was replaced with fresh complete RPMI and cells were stimulated with 1 ng/mL IL-1β (Biolegend) or conditioned media from damaged tendon tissue. After 4 hours, media was removed and 300μL of cell lysis buffer (Life Technologies) was added to each well. Cell lysates were stored at −20°C for subsequent assessment of RNA expression.

### Cell migration

In order to generate conditioned media from tenocytes to induce migration of T cells, tenocytes were stimulated as previously indicated with IL-1β for 4 hours and then washed twice with PBS. Cells were then kept in fresh complete RPMI media for 18 hours, when media was collected and stored at −20°C.

Peripheral blood mononuclear cells (PBMCs) were obtained from healthy donors’ buffy coats from the Scottish National Transfusion Centre after centrifugation with a density gradient (Ficoll-Paque PLUS, GE Healthcare) and CD3+ cells were isolated using negative selection (Stemcell Technologies, Canada). T cells were pre-activated overnight with antibodies against CD3 (aCD3, 1 µg/mL, Biolegend) and CD28 (aCD28, 2 µg/mL, Biolegend). A transwell membrane system (5 µM, Corning) was used to assess the migration of 0.5×10^6^ T cells per well placed in the upper chamber. Conditioned media from tenocytes was diluted to 80% in complete RPMI. After 4 hours, cells migrating to the lower chamber were counted with a haemocytometer and stained for FACS analysis.

### Coculture of tenocytes and T cells

25 000 tenocytes were cocultured with 250 000 T cells (1:10 ratio) obtained after CD3 positive selection (Miltenyi Biotec, Germany) from buffy coats, comparing direct contact between tenocytes and T cells with the effect of soluble factors in cocultures in which a transwell membrane (0.4 µM, Corning) was used to keep both populations apart. After 48 hours, T cells were removed and stained for analysis of the activation marker CD69 by FACS. Supernatants were stored for quantification of IFN-γ by ELISA (Invitrogen, California, USA). Tenocytes were washed twice with PBS in order to remove T cells and then lysed for analysis of gene expression.

### Assessment of T cell activation by FACS

Surface expression of CD69 in T cells was used as a marker of T cell activation. T cells obtained from coculture experiments were transferred to 5 mL polystyrene tubes and washed with PBS. Viability staining was performed with efluor 506 viability dye (Thermo Fisher) for 15 min at 4°C in the dark. After one wash with FACS buffer (PBS+2% FBS), cells were stained for 30 min at 4°C in the dark with the following antibodies: anti-CD3 Pacific Blue, anti-CD4 PE-Cy7, anti-CD8 APC-Cy7, anti-CD69 APC (Biolegend). After incubation, cells were washed once more and fixed with Cell Fix (Becton Dickinson) for 20 min at room temperature. Samples were run in an LSRII flow cytometer (BD Biosciences) and results analysed with FlowJo software.

### Assessment of gene expression

Cells were placed in lysis buffer containing 1% of β-mercaptoethanol and a column system was used for RNA isolation according to manufacturer’s instructions (Life Technologies). Quantification and quality assessment of RNA was performed by spectrophotometry (Nanodrop 2000, Invitrogen, California, USA), cDNA was prepared with High Capacity cDNA reverse transcription kit (Invitrogen) and diluted 1 in 5 using RNase-free water. Real time PCR was performed in duplicate for each sample using SYBR green Master mix (Applied Biosystems, California, USA) and results were normalised using 18S as housekeeping gene. Primers (Integrated Technologies, Belgium) are listed in table 2.

**Table 2:** Table of primers

Gene	Primers
*18S*	5′-GTA ACC CGT TGA ACC CCA TT-3′ (F) 5′-CCA TCC AAT CGG TAG TAG CG-3′ (R)
*IL1B*	5′-CAC CTG TAC GAT CAC TGA ACT G-3′ (F) 5′-AAC ACC ACT TGT TGC TCC ATA-3′ (R)
*IL6*	5′-CAC TCA CCT CTT CAG AAC GAA T-3′ (F) 5′-GCT GCT TTC ACA CAT GTT ACT C-3′ (R)
*IL8*	5′-GTG CAT AAA GAC ATA CTC CAA ACC-3′ (F) 5′-GCT TTA CAA TAA TTT CTG TGT TGG C-3′ (R)
*CCL2*	5′-CTC AGC CAG ATG CAA TCA ATG-3′ (F) 5′-TGC TGC TGG TGA TTC TTC TAT-3′ (R)
*CCL5*	5′-GCT GCT TTG CCT ACA TTG C-3′ (F) 5′-CTT TCG GGT GAC AAA GAC GA-3′ (R)
*CXCL10*	5′-CAC GTG TTG AGA TCA TTG CTA C-3′ (F) 5′-CCT TGC TAA CTG CTT TCA GTA AAT-3′ (R)
*ICAM1*	5′-AGC TTC TCC TGC TCT GCA A-3′ (F) 5′-GGG CCA TAC AGG ACA CGA A-3′ (R)
*COX2*	5′-CAA ATT GCT GGC AGG GTT G-3′ (F) 5′-GGT CAA TGG AAG CCT GTG ATA-3′ (R)
*COL1A*	5′-CAA TGC TGC CCT TTC TGC TCC-3′ (F) 5′-CAC TTG GGT GTT TGA GCA TTG-3′ (R)
*COL3A*	5′-TAT CGA ACA CGC AAG GCT GTG-3′ (F) 5′-GGC CAA CGT CCA CAC CAA ATT-3′ (R)

### Statistical analysis

Data are showed as mean±SEM. Normality of samples was assessed using Shapiro-Wilks, D’Agostino and Pearson or Kolmogorov-Smirnov tests, depending on the sample size, as provided by GraphPad Prism software. In tenocyte stimulations, statistical analysis was performed using paired Student’s t-test or Wilcoxon matched-pairs signed rank test depending on normality. Friedman test with Dunn’s multiple comparison test was used for percentage of migration and one-way analysis of variance (ANOVA) and Dunnett’s multiple comparison test for CD4 and CD8 data in migration experiments; in both cases taking media condition as control. For coculture experiments CD69 analysis was performed also with one-way ANOVA but Tukey’s multiple comparisons test to allow comparison between all groups and for IFN-γ analysis Friedman’s test with Dunn’s multiple comparisons test due to lack of normality of data. Finally, for analysis of gene expression in cocultures, two-way ANOVA with Sidak’s multiple comparisons test was used. In all analyses p<0.05 was considered statistically significant.

## Results

### Transcriptomic analysis and spatial distribution of T cells in tendinopathic tissue

**Figure 1:**
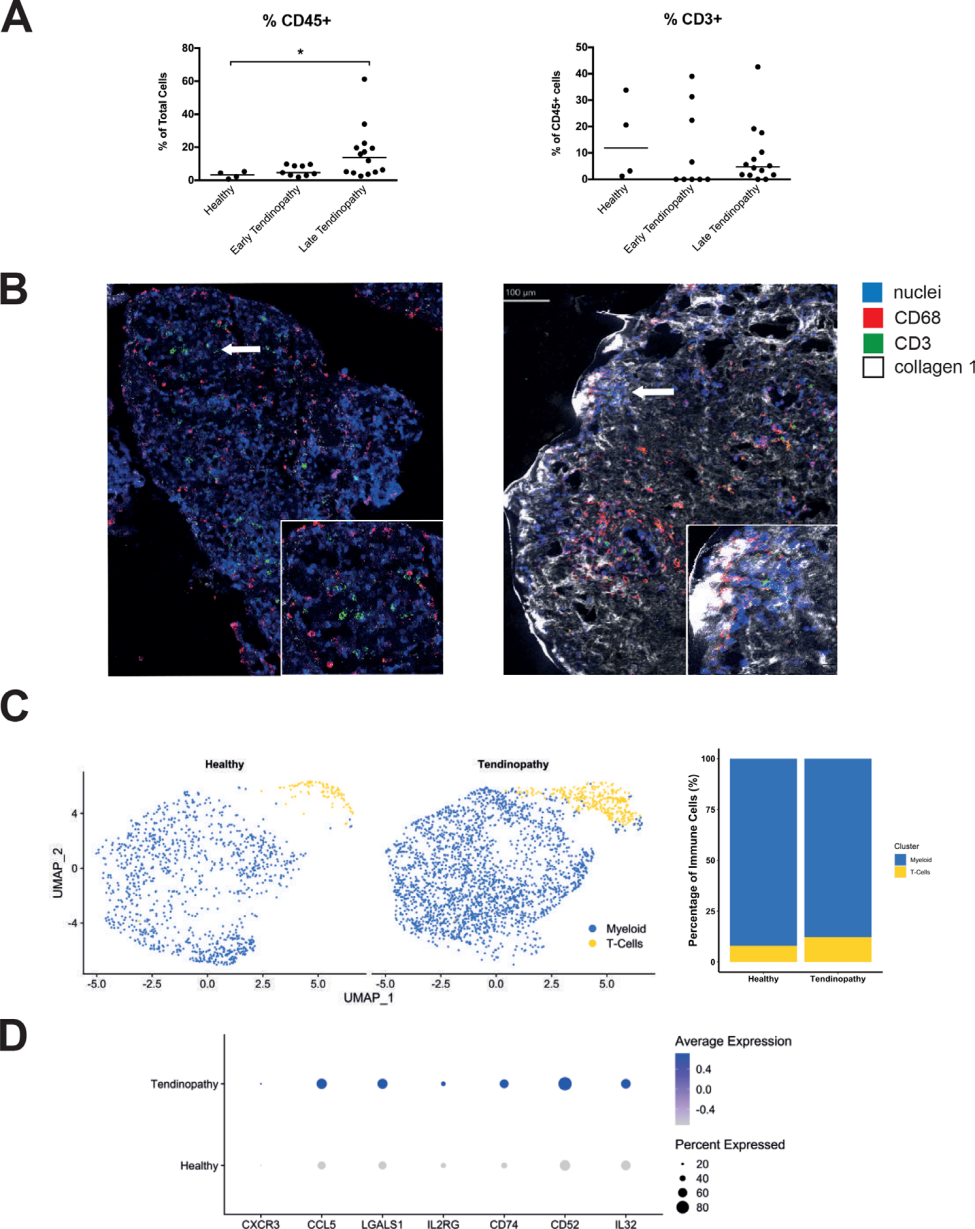
Presence of T cells in tendinopathy. (A) Flow cytometry analysis showing percentage of immune cells (CD45+) and proportion of CD3+ cells in healthy, subscapularis (early tendinopathy) or supraspinatus (late tendinopathy) tendon tissues, n>4, *p≤0.05. (B) Imaging mass cytometry images obtained from analysis of representative supraspinatus tendon sections. Small squares show magnification of the area where T cells and tenocytes interact (white arrow). (C) UMAP of immune cells from tendon tissue following single cell sequencing data from five normal tendons (k=1110) versus four supraspinatus tendons (k=2568). Bar graph shows the percentage of myeloid and T cell populations from total immune cells. (D) Dot plot displaying expression of genes associated with T cell activation from the T cell population in the tendon.

First, we assessed the presence on T cells in tendinopathy. Torn supraspinatus (late tendinopathy) or intact subscapularis (early tendinopathy) biopsies from patients with rotator cuff tears were analysed by FACS. CD45+ immune cells were more frequent in late tendinopathy (16.34%±4.23 %, mean±SEM) compared with control healthy tendon (3.18%±1.03%, mean±SEM) (figure 1A). As previously described myeloid (CD64+) were the predominant immune cell in tendinopathic tissue (online supplemental figure 1B). Interestingly, T cells were present in healthy tendon, and this population increased in tendinopathic tissues (figure 1A) suggesting the potential for an adaptive response throughout the spectrum of tendon disease.

Accordingly, we employed IMC to map the geographical location of immune cells in tendinopathic tissues (figure 1B and online supplemental figure 2B). Spatially there was no consistent evidence of immune cell (T cells CD3+ or macrophages CD68+) clustering in tendinopathic tissue. Both CD3+ and CD68+ cells were primarily interspersed adjacent to tenocytes (Col1high) in tendinopathic tissue suggesting tendon immune cell interactions. Further neighbourhood analysis of this data demonstrated that both T cells and macrophages were present around Col1a positive cells (online supplemental figure 2C).

The presence of T cells in tendon tissue was also assessed by single cell RNA-sequencing from healthy and tendinopathic tendon. Once again, T cells were detected in both healthy and diseased tendon (figure 1C and online supplemental figure 1D). The proportion of T cells in the total immune compartment of tendinopathic tissues (12.23%) was greater than in normal tendon (7.93%) (figure 1C). Furthermore, T cells from tendinopathic tissue had greater expression of a number of genes associated with T cell activation such as *CXCR3*, *CCL5*, *LGALS1* (Galectin-1), *IL2RG*, *CD74*, *CD52* and *IL-32* 27–31 (figure 1D). We also observed genes associated with residency (increase in *TGFB1, NR4A1, PRDM1* and decrease of *KLF2*)^32^ in T cells from healthy tendon and active proliferation (*MKI67, PTTG1*) in diseased tissue (online supplemental figure 3).

10.1136/annrheumdis-2020-219335.supp3 Supplementary data

Taken together, these data demonstrate the presence of T cells in normal tendon with greater proportion present in tendinopathy associated with an overall inflammatory response in damaged tendon which may be due to proliferation, migration or a combination of both.

### Activated tenocytes upregulate inflammatory cytokines and chemokines involved in T cell recruitment

**Figure 2:**
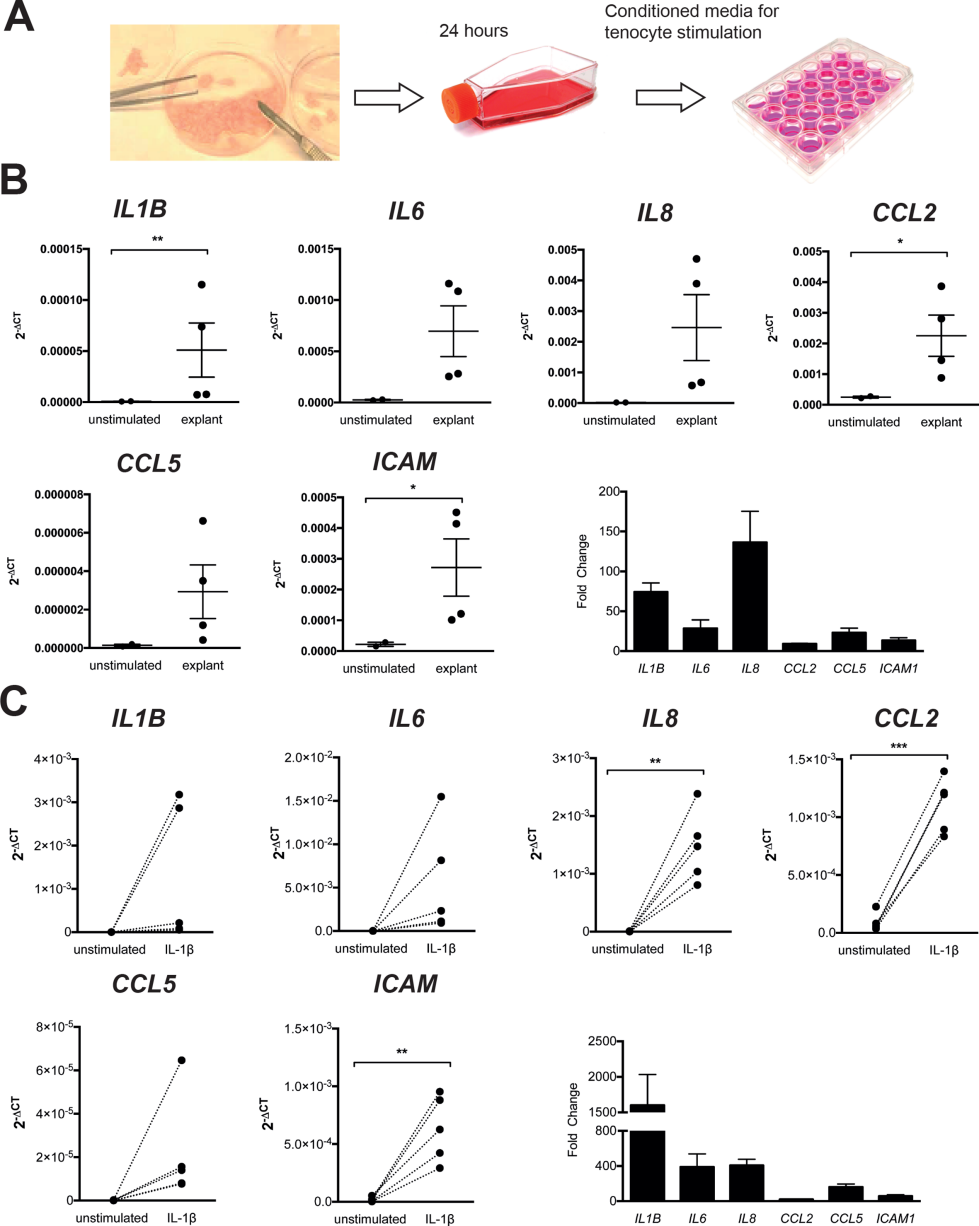
Tenocytes in the context of damage upregulate inflammatory cytokines, chemokines and adhesion molecules. (A) For the obtention of conditioned media from damaged tendon, healthy tendon tissue was cut and transferred into a cell culture flask with complete RPMI for 24 hours. One millilitre of this conditioned media containing products from damaged tissue was then stored and used to stimulate healthy tenocytes. (B) Changes in gene expression of tenocytes from two different healthy tendons after 4-hour stimulation with four different explant conditioned media, graphs show mean±SEM. Statistical analysis comparing mean values of explant stimulation in each tenocyte with unstimulated using paired t-test. (C) Healthy tenocytes were stimulated with interleukin-1β for 4 hours (n=5). Statistical analysis using paired t-test or Wilcoxon matched-pairs signed rank test depending on normality. Graphs show 2^−ΔCt^ after normalisation with the house-keeping gene 18S and fold change in gene expression is relative to unstimulated. *p≤0.05, **p≤0.01, ***p≤0.001, ****p≤0.0001.

Damaged human tendon explants represent an ideal model to explore the pathways whereby tendinopathic pathogenesis may be perpetuated. Accordingly, conditioned media obtained from exogenously damaged healthy hamstring tendon was used to stimulate healthy primary tenocytes (figure 2A). After 4 hours, we observed upregulation in gene expression of inflammatory cytokines (*IL1B*, *IL6*), chemokines (*IL8*, *CCL2* and *CCL5*) and adhesion molecules (*ICAM1*) involved in the recruitment of immune cells (figure 2B). The same effect was observed after activation of tenocytes with IL-1β, which also enhanced the transcription of chemokines capable of T cell recruitment, such as *CCL5* 33 (figure 2C). Thus, the presence of tendon damage is able to activate adjacent tenocytes, rendering primary human tenocytes permissive to T cell recruitment and activation.

### Tenocytes induce T cell migration

**Figure 3:**
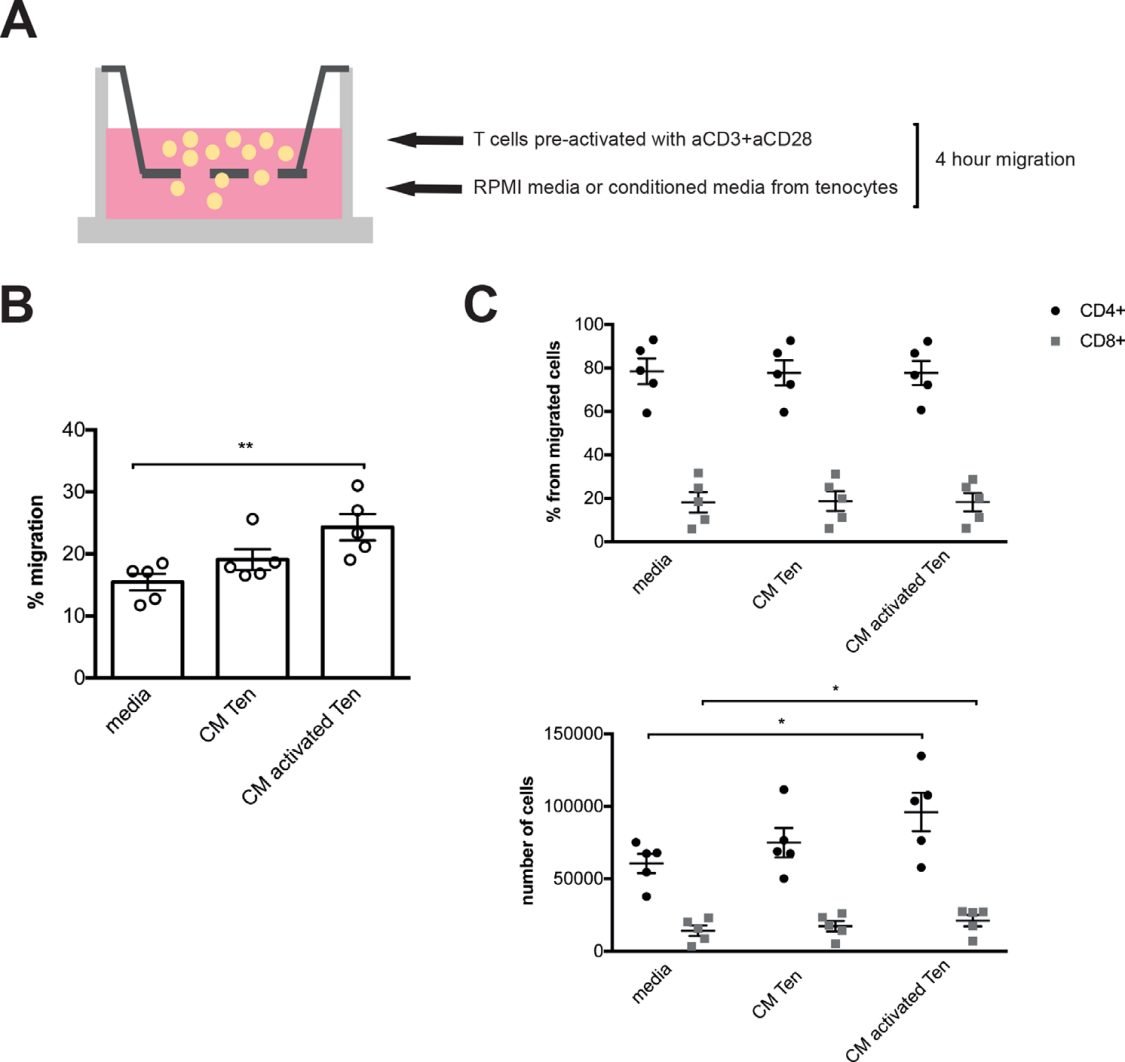
Conditioned media (CM) from activated tenocytes promotes the migration of T cells. (A) T cells were preactivated overnight with anti-CD3 (1 µg/mL) and anti-CD28 (2 µg/mL) antibodies. A transwell membrane system was then used to assess the migratory effect of CM from tenocytes with or without previous activation with interleukin-1β on these activated T cells. (B) Percentage of T cells that migrate through the transwell system after 4 hours. (C) Assessment of CD4+ and CD8+ populations (percentage from total cells that migrate and absolute number of cells) on the migratory fraction by flow cytometry. Data are shown as mean±SEM. Results from two independent experiments with a total of five different tenocyte conditioned media. Statistical analysis using one-way analysis of variance (ANOVA) and Dunnett’s multiple comparison test for CD4 and CD8 data and Friedman test with Dunn’s multiple comparison test for percentage of migration; in both cases taking media condition as control; *p≤0.05, **p≤0.01, ***p≤0.001, ****p≤0.0001.

We next interrogated the effect of activated tenocytes on T cell migration using a transwell system. As in vivo activation of T cells preferentially takes place in regional lymph nodes before subsequent migration into tissue, T cells were preactivated overnight with anti-CD3 and anti-CD28 antibodies and then loaded on top of a transwell membrane, while conditioned media from tenocytes with or without previous activation with IL-1β was placed below the membrane (figure 3A). After 4 hours, the number of migrated T cells was counted (figure 3B) and the proportion of CD4+ and CD8+ cells analysed by FACS (figure 3C). Supernatants from tenocytes, particularly after activation, promoted the migration of T cells as shown by an increase in the percentage of migration (negative control 15.45±1.35, conditioned media from resting tenocytes 19.07±1.68, conditioned media from activated tenocytes 24.3±2.13, data expressed as media±SEM, negative control vs activated tenocytes p=0.003) without preferential recruitment of CD4+ or CD8+ cell subset proportions. This supports the notion that after tissue damage, tenocytes produce chemokines that recruit polyclonal T cells into tendon.

### Direct contact of tenocytes with T cells results in further activation of T cells

**Figure 4:**
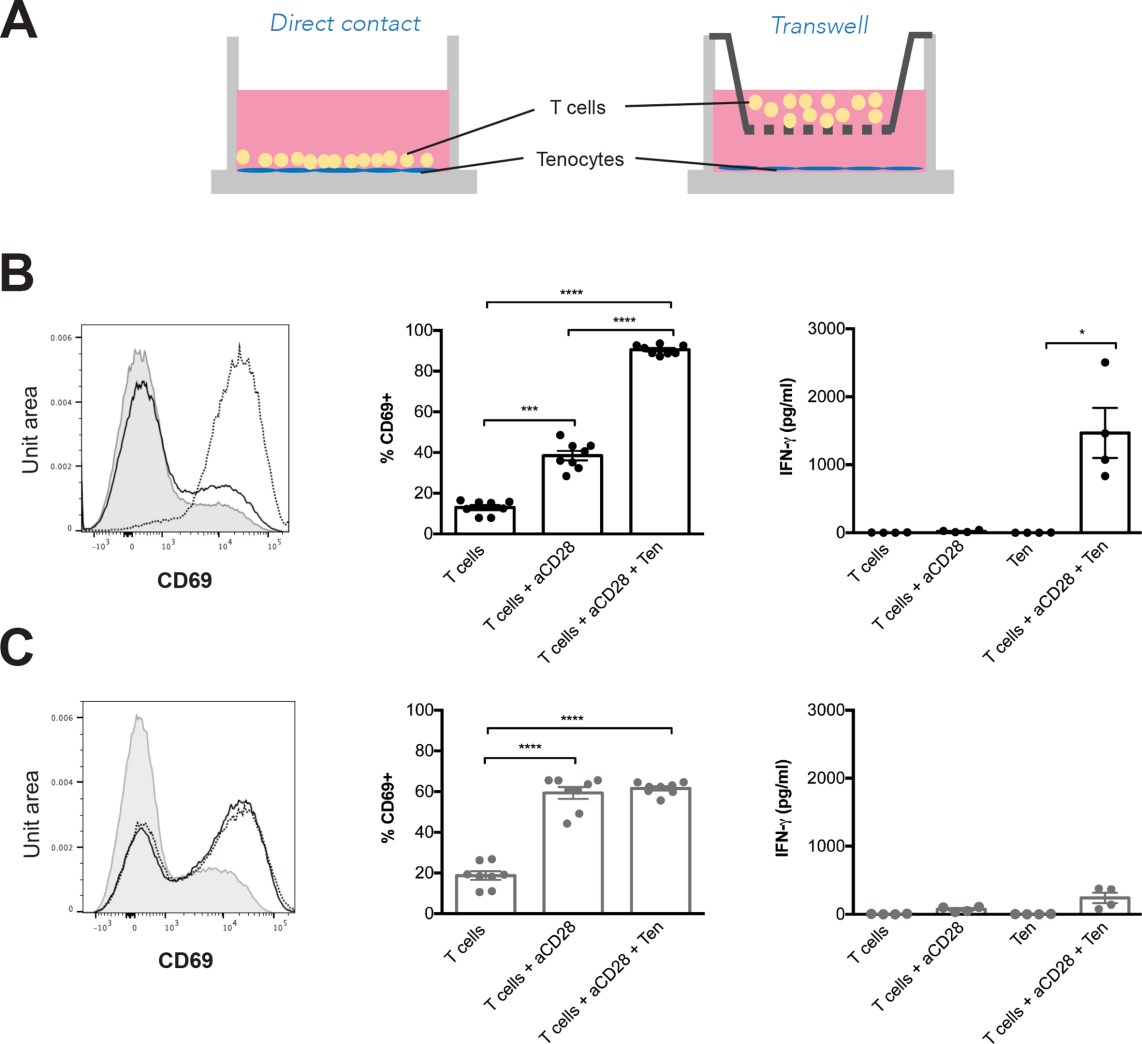
Direct contact of T cells with tenocytes further increases T cell activation. (A) Two different systems were used to coculture tenocytes and T cells (1:10 ratio) for 48 hours; in direct cocultures tenocytes and T cells were in the same well in contact with each other, whereas on the transwell system a membrane was placed between both populations. T cell activation was assessed by CD69 expression by flow cytometry (FACS) and interferon (IFN)-γ production in supernatants by ELISA in both direct (B) and transwell (C) cocultures. Histograms show CD69 expression in unstimulated T cells (grey line), T cells with anti-CD28 (black line) and T cells with anti-CD28 cocultured with tenocytes (dotted line). Results from four independent experiments, each one with a different healthy T cell donor. A total of six different healthy control tenocytes were used. IFN-γ was quantified in two of the experiments. Graphs show data as mean±SEM, statistical analysis using one-way analysis of variance (ANOVA) and Tukey’s multiple comparisons test or FACS data and Friedman’s test with Dunn’s multiple comparisons test for IFN-γ due to lack of normal distribution, *p≤0.05, **p≤0.01, ***p≤0.001, ****p≤0.0001.

To assess if tenocytes can further amplify the inflammatory response, we interrogated their ability to maintain a T cell activation state. T cells isolated from PBMCs were cocultured with healthy explanted tenocytes with or without a transwell membrane, the latter to assess the requirement for cell–cell membrane interactions (figure 4A). We used two methods for isolation of T cells from peripheral blood, negative or positive selection. We also tested the contribution of antigen presenting cells (APC) to the system by adding aCD28. After 48 hours, T cells were harvested and CD69 expression and IFN-γ production were used to assess T cell activation. We observed that coculture of T cells with tenocytes lead to significant increase in activation of T cells as assessed by increased CD69 expression and IFN-γ release, especially when T cells were isolated by positive selection (figure 4 and online supplemental figure 4A). This effect primarily required cell contact between T cells and tenocytes, since it was substantially reduced by transwell cell separation (mean percentage CD69+ cells±SEM; direct contact T cells 12.94±1.2, T cells with aCD28 38.45±2.3, T cells with aCD28 and tenocytes 90.51±0.83 versus transwell system T cells 18.74±2.11, T cells with aCD28 59.39±2.888, T cells with aCD28 and tenocytes 61.63±1.036). Interestingly, this required the presence of anti-CD28, as in its absence IFN-γ levels were undetectable (online supplemental figure 5). This observation may suggest that T cells preactivated in the lymph node by APC such as dendritic cells migrate to the damaged tendon tissue where they are further activated after the contact with tenocytes or alternatively are activated by CD80+ or CD86+ myeloid cells in the tendon.

10.1136/annrheumdis-2020-219335.supp5 Supplementary data

### Activated T cells induce the expression of inflammatory cytokines and chemokines in tenocytes

**Figure 5:**
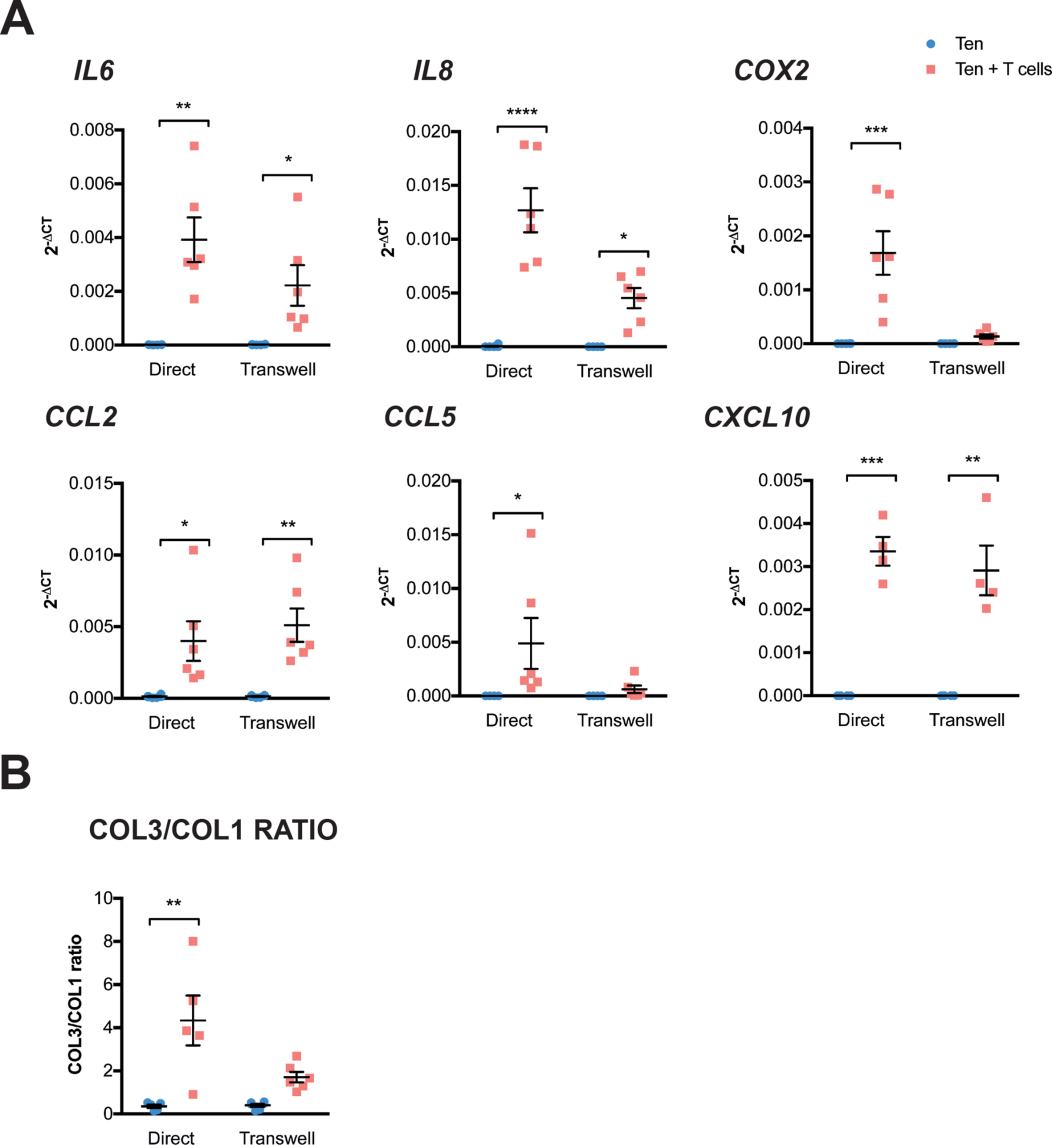
Differential effect of activated T cells on tenocytes. (A) Changes in gene expression of tenocytes after coculture with T cells+anti-CD28 for 48 hours with or without the presence of a transwell membrane to prevent cell contact. Graphs show mean±SEM of 2^−ΔCt^ values after normalisation with the house-keeping gene 18S. (B) Ratio between 2^−ΔCt^ values of COL3 and COL1. Results from three independent experiments involving three different T cell donors and four different tenocyte donors. Statistical analysis using two-way analysis of variance (ANOVA) with Sidak’s multiple comparisons test, *p≤0.05, **p≤0.01, ***p≤0.001, ****p≤0.0001.

We finally interrogated the reciprocal effect that infiltrating T cells may exert on the stromal compartment by analysing changes in gene expression of tenocytes cocultured with activated T cells. After coculture with T cells, we observed an upregulation of inflammatory mediators (*IL-6, COX2*) and chemokines (*IL-8, CCL2, CCL5, CXCL10*) (figure 5A) compared with tenocytes alone. This was particularly evident following direct contact between tenocytes and T cells. Exceptions were *CCL2* and *CXCL10* that were equally upregulated in the presence of the transwell membrane. Coculture of tenocytes and T cells also modulated collagen production, increasing the COL3/COL1 ratio. This effect on the stroma required T cells to be activated, as negative selection of lymphocytes from PBMCs resulted in less activation of T cells when cocultured with tenocytes and no effect on the gene expression of tenocytes (online supplemental figure 4B).

10.1136/annrheumdis-2020-219335.supp4 Supplementary data

## Discussion

**Figure 6:**
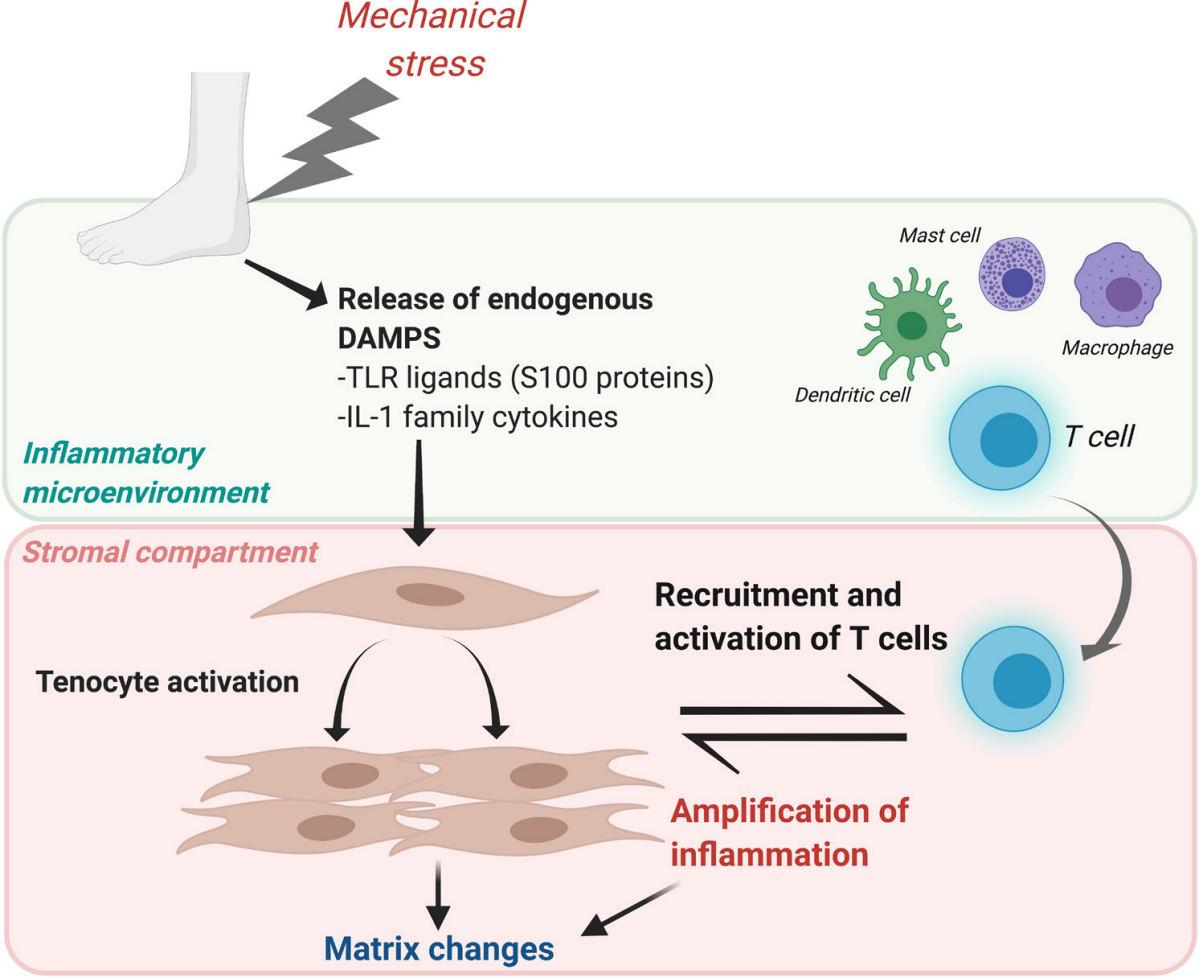
Proposed mechanism of T cell activation after tendon damage. Mechanical stress in the tendon, directly or through the release of endogenous damage-associated molecular patterns (DAMPs) would result in the activation of tenocytes. Once activated, they would produce inflammatory cytokines, chemokines and upregulate adhesion molecules in their surface in order to initiate an immune response to repair the tendon damage. In parallel, antigen presenting cells such as dendritic cells would become activated locally and migrate to the lymph node where they will activate T cells that would migrate to the injured tissue guided by a gradient of chemokines produced after tendon damage. The contact between tenocytes and T cells would further activate these T cells, which in turn amplificate the inflammatory response and promote changes in tendon matrix. IL, interleukin; TLR, toll like receptor.

Emerging evidence supports the role that stromal cell functions extend beyond the maintenance of tissue architecture, playing a key role in choreographing immune responses and thereby defining disease persistence.^34^ In line with this concept, we hypothesised that the interaction between tenocytes and the infiltrating population of immune cells could shape the outcome of the inflammatory response in tendinopathy, leading to resolution or chronicity. Our results demonstrate that in the context of tendon damage, tenocytes upregulate genes involved in inflammation and T cell recruitment and are able to induce migration of activated T cells in vitro. Direct contact of T cells with tenocytes resulted in further activation of these T cells, that in turn upregulated the expression of inflammatory cytokines, chemokines and favoured collagen 3 over collagen 1 in the stromal compartment, creating a feedback loop that may contribute to the establishment of a chronic inflammatory response(figure 6).

Whereas some studies have failed in the past to demonstrate the presence of an inflammatory infiltrate in tendinopathic tissues by immunohistochemistry,^35–39^ we and others have previously reported the presence of T cells and other immune cells.^8^ In the present study we confirmed the presence of T cells in tendinopathy by FACS analysis and single-cell RNA seq of tendon supraspinatus samples from patients with rotator cuff tears undergoing surgery. Our results support the recent observation by Kendal *et al* of the presence of T cells in chronic tendon disease tissue by CITE-seq.^12^ IMC allowed us to assess the infiltration of T cells, both CD4+ and CD8+, supporting the idea of tenocyte–T cell interactions in vivo. Our results could also have implications in other rheumatic disorders such as SpA. In psoriatic arthritis for example, damage or mechanical stress in the tendon has been proposed as the starting point in the development of local inflammation at enthesial sites (attachments of tendons, ligaments and capsules to bones) or enthesitis, that further evolves to joint inflammation or synovitis through what has been referred as ‘synovio-enthesal complex’.^40^ Recently, Watad *et al* reported the presence of T cells in healthy entheseal tissue,^25^ supporting our finding of a population of T cells in healthy tendon tissue. Understanding which factors govern the interaction of T cells with the tendon stroma could help to develop targeted therapies to avoid a chronic inflammatory response.

In order to model the microenvironment that may be present in tendinopathy, we stimulated healthy tenocytes in vitro with conditioned media obtained from tissue explants in which tissue has been previously disrupted, therefore containing a milieu of inflammatory mediators and cell debris mimicking damaged tendon. Stimulation of tenocytes with this conditioned media or IL-1β, a cytokine involved in sterile inflammation, resulted in an upregulation of inflammatory cytokines and chemokines. Stolk *et al,* 41 using conditioned media from anti-CD3 and anti-CD28 stimulated PBMC cultures, observed an upregulation of adhesion (ICAM-1, VCAM-1) and HLA molecules (HLA-ABC, HLA-DR) and IL-6 and when stimulated with IL-1β, tenocytes increased their production of IL-6, IL-8 and CCL2. Together, these results support the role of tenocytes as sensors of damage and their ability to participate in the inflammatory response.

This inflammatory response that takes place after damage usually resolves as infiltrating cells are cleared through induction of apoptosis and phagocytosis by macrophages whereas on the other side chronicity is characterised by the persistence of immune cells. Growing evidence supports the role of an activated or pathogenic stroma in the maintenance of this inflammatory infiltrate and the development of chronic inflammation,^42^ with inflamed tissue expressing inflammatory cytokines, chemokines and adhesion molecules that can lead to migration, retention and survival of leukocytes. After activation in lymph nodes by APC, T cells upregulate CCR5 and CXCR3 allowing them to enter inflamed tissues.^43^ In our system we observed that tenocytes were able to upregulate chemokines involved in T cell recruitment such as CCL5 and CXCL10, that are known ligands of CCR5 and CXCR3, and induce T cell migration, particularly when previously activated with IL-1β. Coculture of tenocytes with T cells resulted in activation of T cells, which notably was contact dependant. To our knowledge this is the first study to investigate the direct interaction between tenocytes and T cells. Previous published work has demonstrated that coculture of macrophages with tenocytes results in an increased IL-6, IL-8 and MCP-1 and CD80 expression on macrophages^41^ and in this case cell contact was not required. In our experiments, cytokines (IL-1β, TNF-α, IL-15) or adhesion molecules such as LFA-1 in T cells and ICAM-1 on tenocytes could have a key role in these cellular interactions.^44 45^

Once activated, T cells also had an effect on tenocytes, with the upregulation of IL-6, IL-8, COX2, CCL2, CCL5 and CXCL10 in tenocytes. Of interest, this effect was observed when T cells were fully activated. This requirement of T cell activation has already been described, as membranes from T cells previously activated with leukophytohaemagglutinin and phorbol myristate acetate were able to induce MMP-1 and PGE2 production in dermal and synovial fibroblasts^46^ and type II collagen stimulated T cells induced the production of IL-17, TNF-α and IL-18 while cocultured with RA synovial fibroblasts.^21^ Of note, the presence of T cells increased collagen 3 proportion, which is one of the pathological features of tendinopathy and that results in a tendon with inferior biomechanics.^1^

There are limitations inherent in our study. Age-related changes within the tendon samples could contribute to the degenerative picture and inflammatory cell expression seen in late tendinopathy. However, the lack of degenerative changes on MRI and arthroscopic examinations suggests that the differences are truly at the cellular level as suggested by our previous work.^14^ Being an in vitro study, we are analysing the interaction between tenocytes and T cells, excluding other factors such as matrix proteins or other immune cells that could also modulate this relationship and the inflammatory process. Accordingly, in our system T cell activation in coculture with tenocytes required the presence of anti-CD28 that in vivo could be provided by other cells such as dendritic cells. Also, we need to take into consideration the effect of an allogenic response in our coculture experiments, although we did not observe differences regarding tenocyte cell death. Further mechanistic investigation in an in vivo tendon injury model could further address the relevance of the adaptive immune response in tendon pathogenesis.

## Conclusion

Our study establishes a previously unrecognised relationship between T cells and stromal cells in human tendon disease. This interaction results in the upregulation of inflammatory cytokines, chemokines and adhesion molecules in activated tenocytes and is also associated with changes in collagen composition, supporting the concept that the adaptive immune response plays a crucial role in a biomechanically associated disease such as tendinopathy. Therefore, selectively targeting T cell signalling in disease provides scope for novel translational strategies in the management of tendon disorders and other enthesial pathologies.

## Data Availability

Data are available upon reasonable request. EGM has access to all the data and data are available upon request.
