# Supplementary material for: Novel self-amplificatory loop between T cells and tenocytes as a driver of chronicity in tendon disease
Source: Ann Rheum Dis. 2021 Mar 10;80(8):1075–85. doi: 10.1136/annrheumdis-2020-219335 (PMC8292554; doi:10.1136/annrheumdis-2020-219335)
Supplement: Supplementary data [file annrheumdis-2020-219335supp001.pdf]

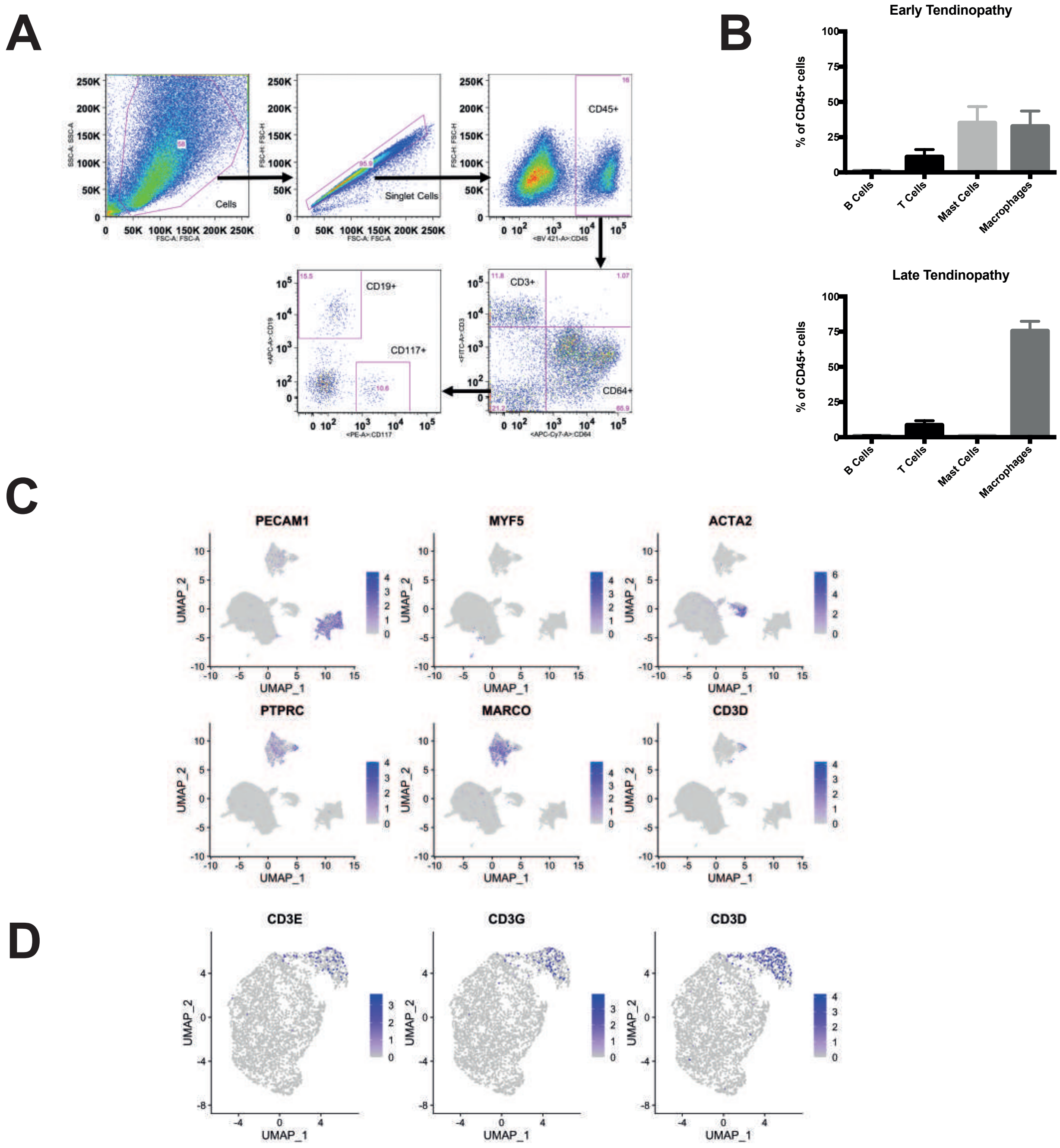

Supplemental Figure 1. Presence of T cells in tendinopathy. (A) Representative FACS plots from disease tissue demonstrating gating strategy to identify immune cell populations in disaggregated tissue. (B) Analysis of the presence of immune cells in tendinopathy by FACS. (C) UMAP of cells isolated from tendon following single cell sequencing identifying endothelial cell (PECAM1), muscle cells (MYF5), myofibroblasts (ACTA2) and immune cells (PTPRC, MARCO, CD3D), data from 5 normal tendons and 4 supraspinatus tendons (k=22124). (C) UMAP immune cells from tendon following single cell sequencing showing the presence of T cells, data from 5 normal tendons and 4 supraspinatus tendons (k=3678).
