# Supplementary material for: Novel self-amplificatory loop between T cells and tenocytes as a driver of chronicity in tendon disease
Source: Ann Rheum Dis. 2021 Mar 10;80(8):1075–85. doi: 10.1136/annrheumdis-2020-219335 (PMC8292554; doi:10.1136/annrheumdis-2020-219335)
Supplement: Supplementary data [file annrheumdis-2020-219335supp002.pdf]

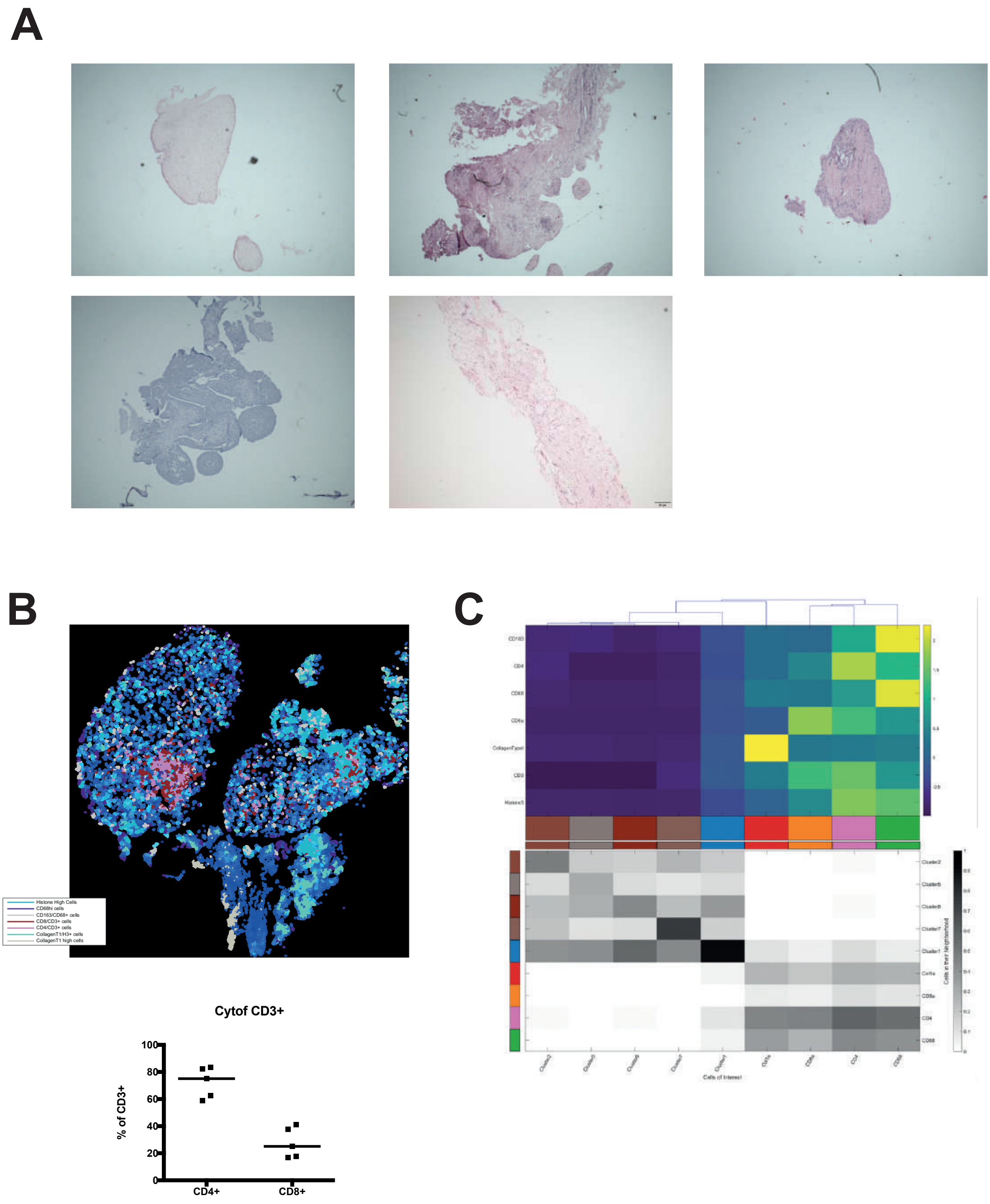

Supplemental Figure 2. Imaging mass cytometry (IMC) of supraspinatus tendon. (A) H&E images of sections used for IMC, 4x magnification. (B) IMC image analysis of supraspinatus tendon section, percentage of CD4+ and CD8+ as measured via IMC, n=5. (C) Representative heatmap showing the z-scored mean marker expression of the panel markers for each PhenoGraph cluster. Interaction heatmap of cluster providing overview of the cell interactions in diseased tissue.

.
