# Supplementary material for: Novel self-amplificatory loop between T cells and tenocytes as a driver of chronicity in tendon disease
Source: Ann Rheum Dis. 2021 Mar 10;80(8):1075–85. doi: 10.1136/annrheumdis-2020-219335 (PMC8292554; doi:10.1136/annrheumdis-2020-219335)
Supplement: Supplementary data [file annrheumdis-2020-219335supp003.pdf]

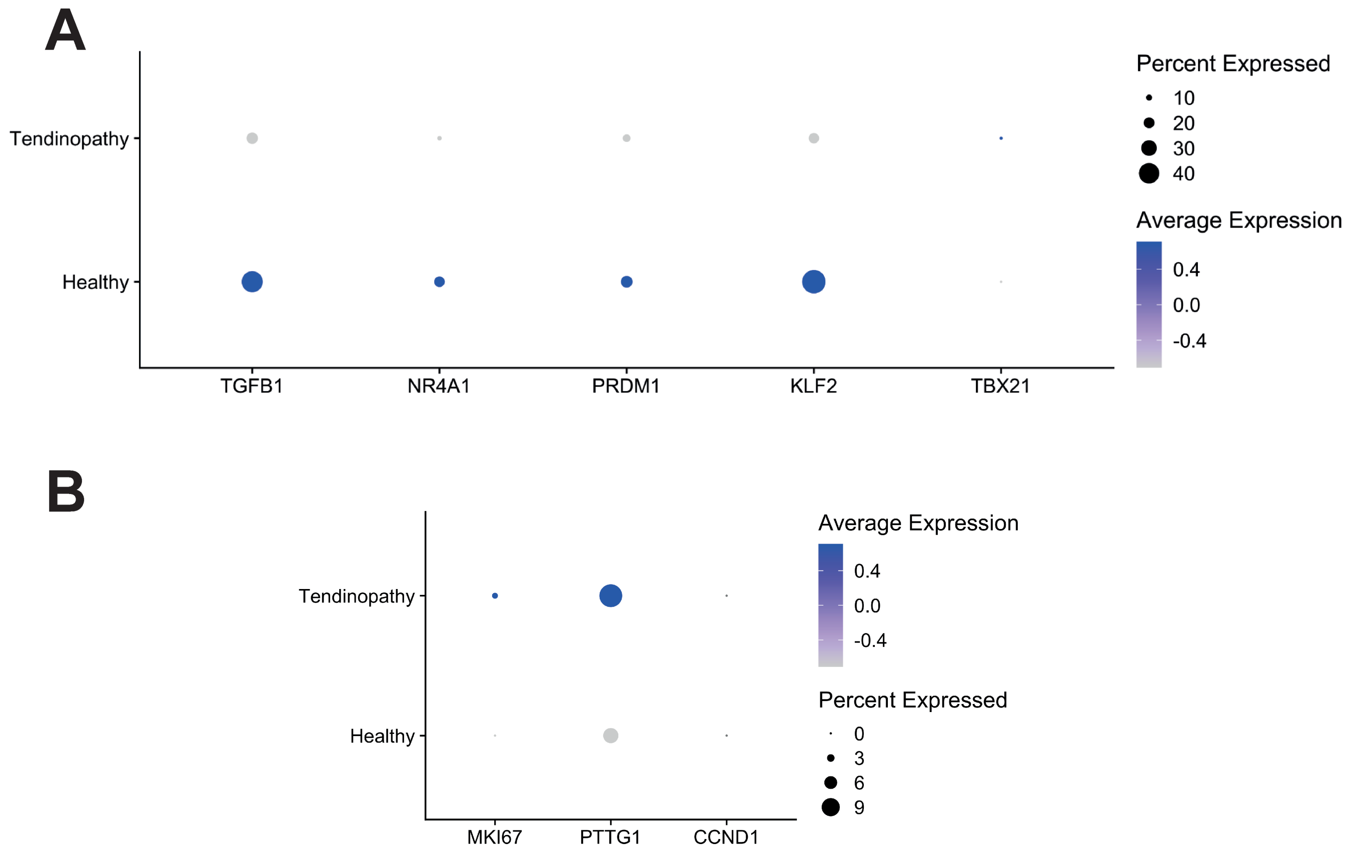

Supplemental Figure 3. Analysis of genes associated with tissue residence and proliferation. (A) Identification of resident T cells in tendinopathy. Dot plot displaying expression of genes associated with resident T cells from the T cell population in the tendon. (B) Identification of proliferating T cells in tendinopathy. Dot plot displaying expression of genes associated with T cell proliferation from the T cell population in the tendon.
