# Supplementary material for: Novel self-amplificatory loop between T cells and tenocytes as a driver of chronicity in tendon disease
Source: Ann Rheum Dis. 2021 Mar 10;80(8):1075–85. doi: 10.1136/annrheumdis-2020-219335 (PMC8292554; doi:10.1136/annrheumdis-2020-219335)
Supplement: Supplementary data [file annrheumdis-2020-219335supp005.pdf]

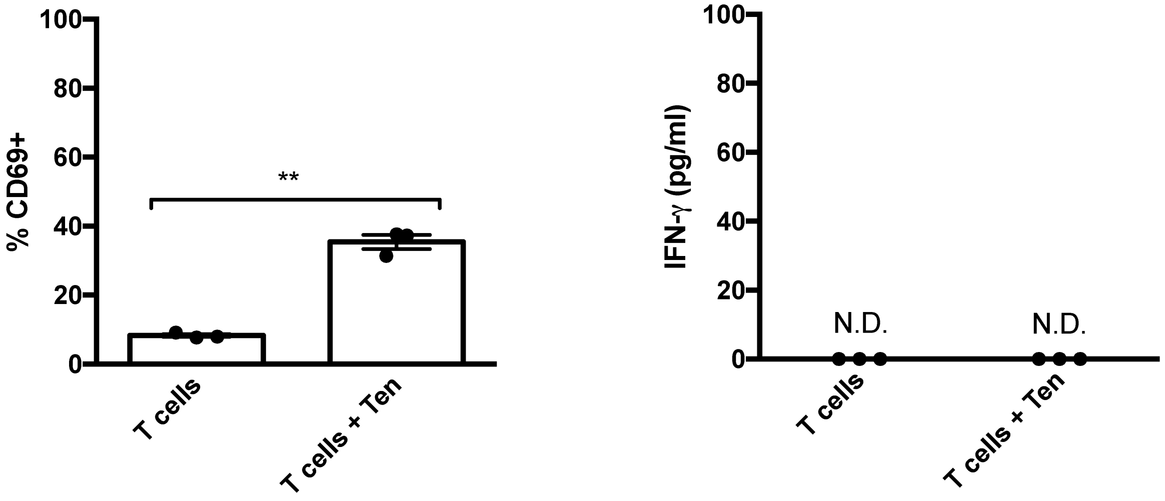

Supplemental Figure 5. Activation of CD3+ T cells in co-culture with tenocytes without the presence of anti-CD28. Cells surface expression of CD69 (A) and IFN-ɣ production (B) in T cells co-cultured for 48h with tenocytes in the absence of anti-CD28 antibody. Results from one experiment with 3 different tenocyte donors and one T cell donor. Graphs show data as mean+/- SEM, statistical analysis using paired t test, (*) $p \leq 0.05$, (**) $p \leq 0.01$, (***) $p \leq 0.001$.
