# Supplementary material for: Novel self-amplificatory loop between T cells and tenocytes as a driver of chronicity in tendon disease
Source: Ann Rheum Dis. 2021 Mar 10;80(8):1075–85. doi: 10.1136/annrheumdis-2020-219335 (PMC8292554; doi:10.1136/annrheumdis-2020-219335)
Supplement: Supplementary data [file annrheumdis-2020-219335supp004.pdf]

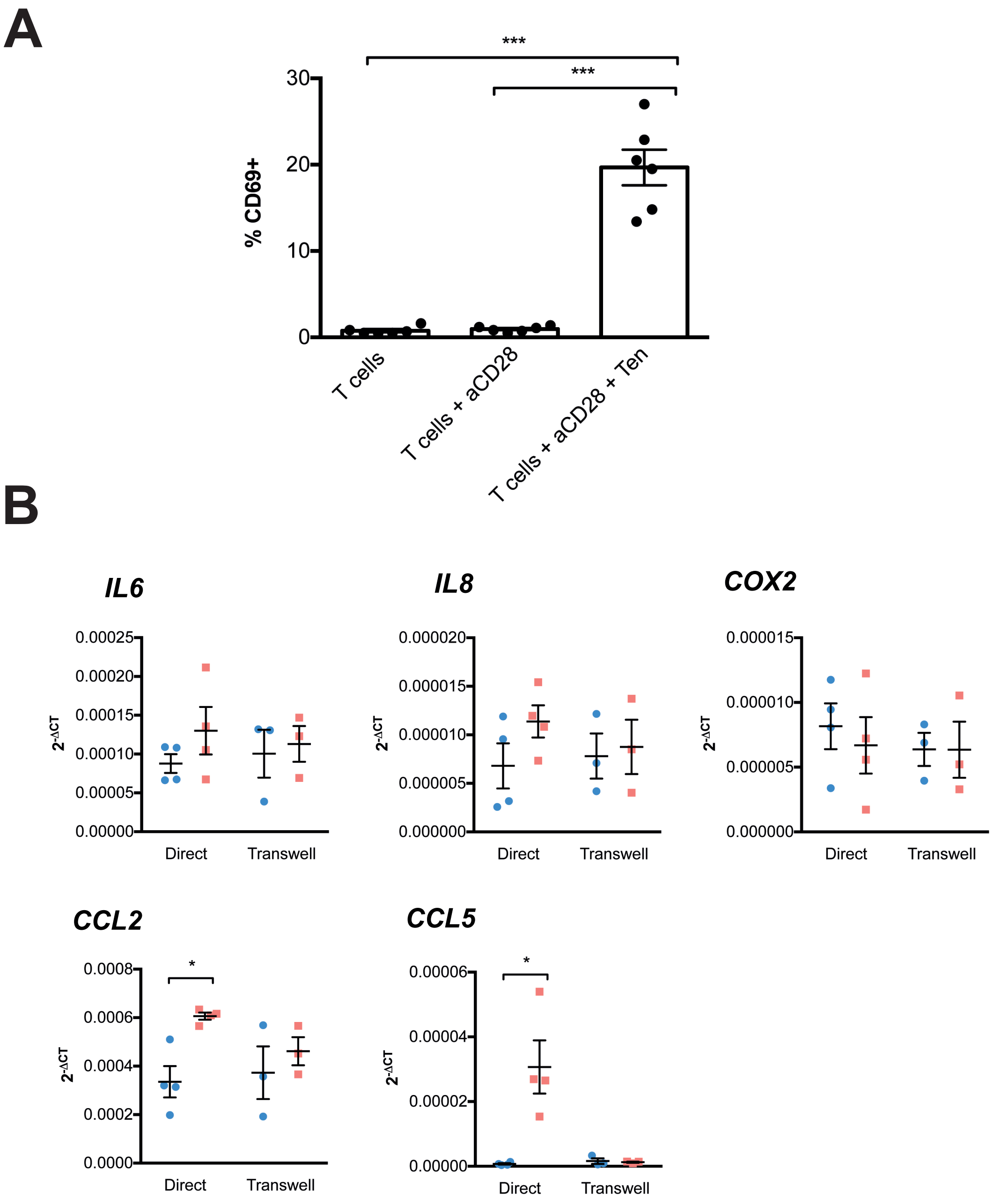

Supplemental Figure 4. Activation of CD3+ T cells isolated with negative selection in co-culture with tenocytes. Percentage of CD69+ cells after 48 hours of culture with or without tenocytes assessed by FACS (A) and changes in transcripts on tenocytes (B) in these co-cultures. T cells were isolated from healthy controls PBMCS using a negative selection method. Results from 3 independent experiments, each one with one T cell donor and 2 tenocyte donors. Graphs show data as mean +/- SEM, statistical analysis using one-way ANOVA and Holm-Sidak's multiple comparisons test for CD69 and 2-way ANOVA with Sidak's multiple comparisons test for gene expression analysis in two independent experiments, (*) $p \leq 0.05$, (**) $p \leq 0.01$, (***) $p \leq 0.001$, (****) $p \leq 0.0001$.
